# Cognitive trajectories and dementia risk in patients with schizophrenia spectrum versus affective disorders

**DOI:** 10.1017/S0033291725101864

**Published:** 2025-09-30

**Authors:** Kathy Y. Liu, Gayan Perera, Robert Howard, Christoph Mueller

**Affiliations:** 1 Division of Psychiatry, University College London, London, UK; 2 Department of Psychological Medicine, Institute of Psychiatry, Psychology & Neuroscience, King’s College London, London, UK; 3 South London and Maudsley NHS Foundation Trust, London, UK

**Keywords:** schizophrenia, depression, affective, cognitive disorder, dementia, aging

## Abstract

**Background:**

Schizophrenia spectrum disorders confer an increased and earlier dementia diagnosis risk, but the relative timing and course of cognitive decline compared to individuals with affective disorders is unclear.

**Methods:**

This retrospective study used de-identified electronic patient records to compare cognitive trajectories from the first recorded MMSE, representing the earliest cognitive concerns in relation to a possible dementia syndrome, and subsequent dementia risk between patients with a schizophrenia spectrum and primary affective disorder diagnosis. Patients had at least two MMSE scores recorded at least 6 months apart. We examined annual MMSE change from the first recorded MMSE, dementia risk, dementia subtypes, and rates of dementia assessment and treatment.

**Results:**

Compared to affective disorders (*n* = 2,264; 71.1 years), schizophrenia spectrum disorders (*n* = 1,217; 65.0 years) showed earlier initial MMSE scores (by 6.1 years, 95% CI = 5.2–7.0), earlier dementia diagnoses (by 2.3 years, 95% CI = 0.9–3.7) but lower dementia risk (adjusted HR = 0.81; 95% CI = 0.69–0.95). Cognitive decline rates and dementia subtype diagnoses did not differ between affective and schizophrenia spectrum disorders, but it took longer for schizophrenia spectrum disorder patients to receive a dementia diagnosis (5.6 vs. 4.4 years). Anti-dementia medication was less likely to be prescribed in patients with schizophrenia versus depression.

**Conclusions:**

Cognitive concerns in older individuals with schizophrenia spectrum disorders arise from around 63 years and are associated with earlier dementia risk versus older individuals with affective disorders. Findings emphasize the importance of targeted dementia prevention and treatment strategies in these individuals and the need to reduce the existing inequity of access to dementia services.

## Introduction

Schizophrenia spectrum disorders, including schizophrenia, schizoaffective disorder, delusional disorder, and schizophreniform disorder, are primary psychotic disorders defined mainly by the presence of persistent psychotic symptoms (i.e., delusions and/or hallucinations with no insight) (Arciniegas, 2015). These conditions, particularly schizophrenia, have been associated with a 2–3 times higher risk (Cai & Huang, 2018; Miniawi, Orgeta, & Stafford, 2022) and an earlier onset (Richmond-Rakerd, D’Souza, Milne, Caspi, & Moffitt, 2022; Stroup et al., 2021) of developing dementia, compared to individuals without these disorders. Precise causes of increased dementia risk are unclear, but as most of the cognitive impairment associated with schizophrenia-related disorders is present at illness onset (Velthorst et al., 2021), these individuals are subsequently closer to crossing a clinical threshold that warrants a dementia diagnosis (Kirkpatrick, Messias, Harvey, Fernandez-Egea, & Bowie, 2008). Cognitive dysfunction in schizophrenia has been conceptualized as ‘primary’ (i.e. arising from schizophrenia-specific neurobiological and neurophysiological alterations) and ‘secondary’ (i.e. the consequence of any other factor) cognitive impairment (Vita, Nibbio, & Barlati, 2024). Baseline cognitive impairment is likely to be due to shared risk factors for poorer cognition and schizophrenia risk, such as genetic factors and increased exposure to environmental factors, including prenatal complications, urbanicity, socioeconomic deprivation, drug/cannabis use, and low educational attainment (McCutcheon, Keefe, & McGuire, 2023).

After illness onset and up to the age of 65 years, cognitive function in schizophrenia spectrum disorders is reported to mainly remain stable and generally follow normal age-related trajectories (Fett et al., 2020; Velthorst et al., 2021). Worse physical health, effects of medications, and sociodemographic factors (Bendayan, Mascio, Stewart, Roberts, & Dobson, 2021; McCutcheon et al., 2023) during mid-life may drive further cognitive decline in older individuals, but cognitive trajectories in relation to these factors have been poorly characterized. Clarification of cognitive trajectories in psychotic disorders in relation to dementia risk in later life (after 60 years) is needed, and the identification of any predictors of dementia risk could potentially lead to targeted prevention and treatment approaches.

It would be relevant to compare cognitive trajectories and dementia risk between schizophrenia spectrum disorders and other mental health conditions, such as bipolar (Diniz et al., 2017) and major depressive disorders (Diniz, Butters, Albert, Dew, & Reynolds, 2013; Singh-Manoux et al., 2017), which are also associated with increased dementia risk and earlier age of dementia diagnosis (Richmond-Rakerd et al., 2022). In contrast to earlier studies that have generally enrolled participants based on age, it would also be informative to examine cognitive trajectories starting from when clinical cognitive concerns first occurred. The Mini-Mental State Examination (MMSE) (Folstein, Folstein, & McHugh, 1975) is a commonly used clinical screening tool to assess cognition when dementia is suspected, and to monitor cognitive changes through longitudinal assessments. A decline of up to a 0.5 MMSE point per year is consistent with normal age-related cognitive decline in older adults (Nagaratnam, Sharmin, Diker, Lim, & Maier, 2022), whereas an annual decline of at least two MMSE points is consistent with AD dementia (Clark et al., 1999; Han, Cole, Bellavance, McCusker, & Primeau, 2000).

This study aimed to investigate cognitive trajectories, from the first recorded MMSE, and subsequent dementia risk in patients with a schizophrenia spectrum or primary affective disorder, and compare findings between these groups. We hypothesized that initial cognitive concerns would arise earlier in schizophrenia spectrum disorders, as these individuals show cognitive impairments before illness onset to a greater degree compared to bipolar disorder and depression. We also expected that, in line with previous studies, this would correspond to an earlier age of dementia diagnosis and higher dementia incidence.

## Methods

### Data source and study cohorts

A clinical register-based and de-identified patient cohort was selected using a Clinical Record Interactive Search (CRIS) (Perera et al., 2016). The CRIS resource renders a de-identified version of the South London and Maudsley (SLaM) National Health Service (NHS) Foundation Trust’s electronic record available for research purposes (Perera et al., 2016) to extract data. SLaM is one of Europe’s largest mental health care providers and serves a population of more than 1.3 million people in four south London boroughs (Lambeth, Lewisham, Southwark, and Croydon) in England. Data from CRIS have been considerably supplemented through natural language processing applications using Generalised Architecture for Text Engineering (GATE) software (Cunningham, Tablan, Roberts, & Bontcheva, 2013). In addition to data recorded in structured fields, this allows the extraction of information from free text fields (e.g. clinical events, correspondence) in the mental health record. The dataset comprised de-identified data for secondary analysis; thus, informed consent was not required. The authors assert that all procedures contributing to this work comply with the ethical standards of the relevant national and institutional committees on human experimentation and with the Helsinki Declaration of 1975, as revised in 2008. All procedures involving human subjects/patients were approved by Oxford Research Ethics Committee C, reference 18/SC/0372, and individual projects were approved by a patient-led oversight committee.

Participants accessing SLaM NHS Foundation Trust services between January 1, 2008, and March 30, 2021, who had a diagnosis of schizophrenia spectrum (schizophrenia, schizotypal, delusional, and other non-mood psychotic) or affective disorders, and at least two MMSE scores documented at least 6 months apart were included. The WHO International Classification of Diseases version 10 (ICD-10) codes used to define these conditions are provided in Table 1. Patients were excluded if a diagnosis of dementia was recorded before or within 3 months of a schizophrenia spectrum or affective disorder diagnosis. Schizoaffective disorders were not included in the schizophrenia spectrum cohort due to the high degree of clinical overlap with affective disorders.

**Table 1.** ICD-10 codes were used to define study cohorts

Cohorts	ICD-10 codes
Schizophrenia spectrum disorders	F20–F29
Schizophrenia	F20
Other schizophrenia spectrum disorder	F21–F29
Primary affective disorders	F30–F39
Depression	F32, F33
Bipolar affective disorder	F30, F31
Other affective disorder	F34, F38, F39
Dementia	F00–F03
Mild cognitive impairment^a^	F06.7

a Used to measure rates of previous diagnoses, but not intended to define a study cohort.

### Outcomes

We identified MMSE scores at up to four time-points in individual patients’ electronic health records: (1) the first recorded MMSE score, (2) the second MMSE score recorded at least 6 months after the first MMSE score, (3) the last recorded MMSE, and (4) for patients with dementia, the last MMSE score recorded before a dementia diagnosis. Mean annual MMSE decline rates were calculated by dividing the change in MMSE score between the first and subsequent MMSE recordings by the duration (in years) between the recordings.

For those who were eventually diagnosed with dementia, we established the dementia subtype diagnosis, whether they were diagnosed in a memory service (as opposed to a mental health service), whether a previous diagnosis of mild cognitive impairment (MCI) had been given, and whether an acetylcholinesterase inhibitor and/or memantine were prescribed at any point after dementia diagnosis.

For Cox regression models, patients were followed from their first recorded MMSE until they received a diagnosis of dementia, or until their last recorded face-to-face contact with SLaM health services, a record of death, or a censoring date on December 20, 2022.

### Covariates

Demographic information included age, sex, ethnicity (White, Black, Asian, other), marital/cohabitation status, and index of multiple deprivations (Noble et al., 2007). The Health of the Nation Outcome Scales (HoNOS) subscales were included to obtain information on mental health, physical health, and functional problems recorded close to the time of the first MMSE score. HoNOS scales are routinely used in UK mental health services and recorded as structured data on the electronic health record (Burns et al., 1999). HoNOS subscale scores for each item range from 0 (no problem) to 4 (severe problem), and to facilitate interpretation, we dichotomized the scores into ‘no or mild problem’ (scores 0–1) and ‘problem present’ (scores 2–4). We identified whether patients were prescribed an antidepressant and/or an antipsychotic, via natural language processing algorithms applied to free text (Mueller et al., 2021), up to 6 months before and after the first MMSE recording.

### Statistical analysis

We used STATA 15 software (Stata Corp LP, College Station, TX) for all analyses. To examine group differences between schizophrenia spectrum and affective disorders, we conducted Mann–Whitney *U* tests for continuous variables (as these were non-normally distributed) and chi-squared tests for categorical variables. We used Cox proportional hazards regression models to compare dementia risk between groups. Models were adjusted for age (at first MMSE score), participant sex, ethnicity, marital/cohabitation status, index of multiple deprivations, and age squared to model a potential non-linear effect of age more accurately. In sensitivity analyses, we compared the schizophrenia and depression groups using the same tests. To identify potential early predictors of dementia risk, particularly within individuals with schizophrenia and schizophrenia spectrum disorders, we applied Cox proportional hazards regression models (adjusted for age, age squared, gender, ethnicity, marital status, and deprivation score) within each cohort separately. Statistical significance was considered at a *p*-value of <0.05 (alpha = 0.05).

To determine if and how our cohorts with at least two MMSE recordings differed from individuals in whom there were no cognitive concerns, we identified age-matched control cohorts of patients with either a schizophrenia spectrum or affective disorder who did not have at least two MMSE scores. Patients with at least two MMSE score points were one-in-three matched to those without, according to 5-year age bands, whereby control patients could be repeatedly assigned to exposed patients.

## Results

We identified 1,217 patients with schizophrenia spectrum disorder diagnoses and 2,264 patients with primary affective disorder diagnoses in the 12-year observation period, who had at least two MMSE scores recorded at least 6 months apart. For the whole cohort in whom there were cognitive concerns, the first two MMSEs were recorded 2.79 (SD 2.71) years apart, and the duration between the first recorded MMSE to the end of follow-up was 6.1 (SD 4.2) years.

### Cohort characteristics

For patients with any schizophrenia spectrum disorder, 727 (59.7%) had a diagnosis of schizophrenia, and 490 (40.3%) had another psychotic disorder. For patients with an affective disorder, 1,822 (80.5%) had a diagnosis of depression, 333 (14.7%) had a diagnosis of bipolar affective disorder, and the remaining 109 (4.8%) had another affective disorder diagnosis.

Compared to patients with an affective disorder, patients with a schizophrenia spectrum disorder were more likely to be male, Black, living in more deprived areas, and less likely to be married or cohabiting (Table 2). According to HoNOS scores at the time of the first MMSE recording, a higher proportion of individuals with schizophrenia spectrum disorders experienced agitated behavior, substance use, and hallucinations and/or delusions, whereas patients with affective disorder were more likely to have non-accidental self-injuries, cognitive problems, depressed mood, and physical illness or disability.

**Table 2.** Characteristics of patients with schizophrenia spectrum disorder or affective disorders in whom there were cognitive concerns

	Schizophrenia spectrum (*n* = 1,217)	Affective disorder (*n* = 2,264)	p-value^a^
Diagnosed with dementia (%)	20.6	25.7	**0.001**
Mean age in years (SD)			
At 1st MMSE	65.0 (14.8)	71.1 (12.0)	**<0.001**
At 2nd MMSE	68.0 (14.4)	73.8 (11.7)	**<0.001**
At last MMSE	70.2 (14.9)	75.7 (11.8)	**<0.001**
Other Sociodemographic characteristics			
Female (%)	54.2	62.6	**<0.001**
Ethnicity (%)			**<0.001**
White	50.1	75.7	
Black	40.0	15.3	
Asian	7.2	6.7	
Other	2.7	2.3	
Married or cohabiting (%)	14.7	29.2	**<0.001**
Mean index of multiple deprivations (SD)	27.2 (9.1)	25.8 (9.8)	**<0.001**
Mean MMSE score (SD)			
1st MMSE	24.0 (5.9)	24.4 (5.4)	0.075
2nd MMSE	23.0 (6.2)	23.1 (6.3)	0.606
Last MMSE	21.8 (6.9)	21.8 (7.0)	0.667
Annual MMSE score decline (95% CI)			
1st to 2nd MMSE	−0.35 (−0.59 to −0.12)	−0.56 (−0.75 to −0.37)	0.288
1st to last MMSE	−0.54 (−0.72 to −0.36)	−0.72 (−0.87 to −0.58)	0.136
HoNOS Mental health problems^b^ (%)			
Agitated behaviour	19.7	15.5	**0.002**
Non-accidental self- injury	3.1	9.5	**<0.001**
Substance and/or alcohol use	9.8	7.7	**0.031**
Cognitive problems	27.6	31.3	**0.024**
Hallucination and/or delusions	57.9	14.5	**<0.001**
Depressed mood	17.7	55.5	**<0.001**
HoNOS Physical health and functional problems^b^ (%)			
Physical illness or disability	43.6	54.9	**<0.001**
Problems with activities of daily living	39.8	39.5	0.859
Pharmacotherapy^b^ (%)			
Antidepressant	30.6	70.2	**<0.001**
Antipsychotic	79.1	33.7	**<0.001**

a Mann–Whitney *U* test or chi^2^ test; statistically significant differences highlighted in bold text.

b Around the time of the 1st MMSE recording.

### MMSE score and age differences between schizophrenia spectrum and affective disorders

The first, second, and last MMSE scores were recorded around 6 years earlier in patients with schizophrenia spectrum disorder (at 65, 68, and 70 years of age) compared to those with an affective disorder (at 71, 74, and 76 years of age) (Table 2). There were no significant differences in mean MMSE scores or rates of cognitive decline between schizophrenia spectrum and affective disorder groups. The mean annual decline between the first and last recordings were −0.54 and −0.72 MMSE points for the schizophrenia spectrum and affective disorder groups, respectively.

### Progression to dementia

Of individuals with a schizophrenia spectrum disorder, 251 (20.6%) were subsequently diagnosed with dementia, versus 581 (25.7%) with a primary affective disorder. Correspondingly, a Cox regression model showed patients who had a schizophrenia spectrum disorder had a lower risk of receiving a dementia diagnosis compared to those with an affective disorder (hazard ratio (HR): 0.61; 95% CI: 0.52–0.70; *p* < 0.001), which remained significant after adjusting for potential demographic confounders (HR: 0.81; 95% CI: 0.69–0.95; *p* = 0.010).

For patients who were subsequently diagnosed with dementia, the first and second mean MMSE scores in the schizophrenia spectrum group were recorded around 3–4 years earlier than the affective disorder group (at 72 and 75 vs. 76 and 78 years of age, respectively; Table 3). The last mean MMSE score before dementia diagnosis was (significantly) 1 point lower in the schizophrenia spectrum compared to the affective disorders group. There were no differences in the rates of cognitive decline between the cohorts who were diagnosed with dementia, and annual declines between the first and last recordings were −1.29 and −1.59 MMSE points for the schizophrenia spectrum and affective groups, respectively.

**Table 3.** Characteristics of patients with schizophrenia spectrum or affective disorders in whom there were cognitive concerns and who were diagnosed with dementia

	Schizophrenia spectrum & dementia (*n* = 251)	Affective disorder & dementia (*n* = 581)	*p*-value^a^
Mean age in years (SD)			
At 1st MMSE	72.0 (9.8)	75.5 (9.6)	**<0.001**
At 2nd MMSE	74.5 (9.9)	78.1 (9.4)	**<0.001**
At last MMSE before dementia diagnosis	76.7 (10.0)	79.3 (9.2)	**<0.001**
At last MMSE in record	78.1 (9.6)	80.6 (9.0)	**<0.001**
At dementia diagnosis	77.6 (9.8)	79.9 (9.2)	**0.001**
Other sociodemographic characteristics			
Female (%)	64.1	67.1	0.404
Ethnicity (%)			**<0.001**
White	52.0	74.5	
Black	39.2	15.9	
Asian	7.2	7.8	
Other	1.6	1.7	
Married or cohabiting (%)	18.3	29.8	**<0.001**
Mean index of multiple deprivations (SD)	26.9 (9.1)	24.4 (9.6)	**0.001**
Mean MMSE score (SD)			
1st MMSE	22.8 (6.3)	23.9 (5.5)	0.051
2nd MMSE	21.1 (6.8)	21.2 (6.6)	0.977
Last MMSE before dementia diagnosis	18.7 (6.6)	19.9 (6.4)	**0.019**
Last MMSE in the record	17.6 (7.1)	18.6 (6.9)	0.089
Annual MMSE score decline (95% CI)			
1st to 2nd MMSE	−0.96 (−1.45 to – 0.48)	−1.31 (−1.67 to −0.95)	0.097
1st to last MMSE before dementia diagnosis	−1.38 (−1.75 to −1.00)	−1.51 (−1.81 to −1.21)	0.313
1st to last MMSE	−1.29 (−1.56 to −1.01)	−1.59 (−1.85 to −1.34)	0.248
HoNOS Mental health problems^b^ (%)			
Agitated behavior	13.2	14.7	0.564
Non-accidental self- injury	1.2	6.8	**0.001**
Substance and/or alcohol use	5.3	5.8	0.781
Cognitive problems	32.5	35.1	0.479
Hallucination and/or delusions	59.6	14.6	**<0.001**
Depressed mood	16.8	53.3	**<0.001**
HoNOS Physical health and functional problems^b^ (%)			
Physical illness or disability	46.8	58.7	**0.002**
Problems with activities of daily living	37.6	39.0	0.707
Pharmacotherapy^b^ (%)			
Antidepressant	28.7	69.7	**<0.001**
Antipsychotic	78.1	26.7	**<0.001**
Dementia subtypes (%)			
Alzheimer’s disease	30.3	34.1	0.284
Vascular dementia	23.1	23.6	0.883
Mixed-type dementia (AD and vascular)	19.5	20.3	0.795
DLB	4.8	4.5	0.846
Unspecified or other	22.3	17.6	0.109
Diagnostic services and treatments (%)			
Diagnosis in a memory service	17.9	26.7	**0.007**
Prior diagnosis of Mild Cognitive Impairment	13.2	18.9	**0.042**
Acetylcholinesterase inhibitor prescribed^c^	39.8	43.0	0.392
Memantine prescribed^c^	17.9	19.5	0.608

a Mann–Whitney *U* test or chi^2^ test; statistically significant differences highlighted in bold text.

b Around the time of the 1st MMSE recording.

c Anytime in the patient’s record.

On average, patients with schizophrenia spectrum disorders who were diagnosed with dementia received the diagnosis 5.6 years after their first recorded MMSE, compared to 4.4 years for patients with an affective disorder (Table 3). At the time of dementia diagnosis, the schizophrenia spectrum disorders group was 2.3 years younger than the affective disorders group (77.6 vs. 79.9 years of age). While there were no differences in dementia subtypes and prescribing of anti-dementia medication between the groups, those who were diagnosed with dementia on the background of a schizophrenia spectrum disorder were less likely to have received this diagnosis in a memory clinic or to have previously received a diagnosis of mild cognitive impairment (Table 3).

### Sensitivity analysis of schizophrenia versus depression

Differences in baseline characteristics between the schizophrenia and depression sub-samples were similar to the overall cohort, except that no differences on the HoNOS cognitive problems subscale were detected (Table 4). Mean duration between the first two MMSE scores and mean follow-up time were also similar to the overall cohort, but the rate of cognitive decline (1st to 2nd MMSE and 1st to last MMSE scores) was higher in the depression group. As seen in the larger diagnostic cohorts, a higher proportion of patients with depression (*n* = 487, 26.7%) were diagnosed with dementia compared to patients with schizophrenia (*n* = 142, 19.5%). Correspondingly, Cox regression models showed that patients with schizophrenia had a lower dementia risk versus patients with depression (HR: 0.48; 95% CI: 0.40–0.58; *p* < 0.001), which remained significant after adjusting for age, age-squared, gender, ethnicity, marital status and deprivation (HR: 0.71; 95% CI: 0.58–0.88; *p* = 0.002).

**Table 4.** Characteristics of patients with schizophrenia and depression in whom there were cognitive concerns

	Schizophrenia (*n* = 727)	Depression (*n* = 1,822)	*p*-value^a^
Diagnosed with dementia (%)	19.5	26.7	**<0.001**
Mean age (SD)			
At 1st MMSE (SD)	63.3 (14.8)	72.2 (11.7)	**<0.001**
At 2nd MMSE (SD)	66.4 (14.5)	74.8 (11.5)	**<0.001**
At last MMSE (SD)	68.7 (15.0)	76.5 (11.6)	**<0.001**
Other sociodemographic characteristics			
Female (%)	48.4	63.2	**<0.001**
Ethnicity (%)			**<0.001**
White	47.2	75.6	
Black	41.7	14.9	
Asian	7.8	7.1	
Other	3.3	2.4	
Married or cohabiting (%)	11.6	30.5	**<0.001**
Mean index of multiple deprivations (SD)	27.7 (8.9)	25.9 (9.8)	**<0.001**
Mean MMSE scores (SD)			
1st MMSE	23.5 (6.1)	24.3 (5.5)	**0.004**
2nd MMSE	22.6 (6.5)	22.8 (6.3)	0.479
Last MMSE	21.3 (7.2)	21.5 (7.0)	0.501
Annual MMSE score decline (95% CI)			
1st to 2nd MMSE	−0.30 (−0.61 to 0.00)	−0.65 (−0.87 to −0.44)	**0.013**
1st to last MMSE	−0.55 (−0.77 to −0.33)	−0.82 (−1.00 to −0.65)	**0.012**
HoNOS Mental health problems^b^ (%)			
Agitated behaviour	19.4	13.4	**<0.001**
Non-accidental self- injury	2.9	10.5	**<0.001**
Substance and/or alcohol use	11.0	7.5	**0.005**
Cognitive problems	31.2	32.9	0.402
Hallucination and/or delusions	56.0	12.5	**<0.001**
Depressed mood	16.2	61.0	**<0.001**
HoNOS Physical health and functional problems^b^ (%)			
Physical illness or disability	42.1	56.7	**<0.001**
Problems with activities of daily living	44.5	40.4	0.063
Pharmacotherapy^b^ (%)			
Antidepressant	28.5	75.1	**<0.001**
Antipsychotic	85.6	26.7	**<0.001**

a Mann–Whitney U test or chi^2^ test; statistically significant differences highlighted in bold text.

b Around the time of the 1st MMSE recording.

For patients who were subsequently diagnosed with dementia (Table 5), those with a background of schizophrenia had the first two MMSE scores recorded around 6 years earlier than in those with depression (at 70 and 73 vs. 76 and 79 years of age), and at each recording, MMSE scores were around 1–2 MMSE points lower. There were no significant differences in cognitive decline rates between the groups, and the annual MMSE score change between the first and last recordings were −1.24 and −1.70 MMSE points for the schizophrenia and depression groups, respectively. There were no differences in dementia subtype diagnosed or whether the diagnosis occurred in a memory service, but patients with schizophrenia were significantly less likely to be prescribed an acetylcholinesterase inhibitor or memantine and less likely to have a prior diagnosis of MCI.

**Table 5.** Characteristics of patients with schizophrenia or depression in whom there were cognitive concerns and who were diagnosed with dementia

	Schizophrenia & dementia (*n* = 142)	Depression & dementia (*n* = 487)	*p*-value^a^
Mean age (SD)			
At 1st MMSE	70.3 (10.4)	76.1 (9.6)	**<0.001**
At 2nd MMSE	72.9 (10.4)	78.7 (9.3)	**<0.001**
At last MMSE before dementia diagnosis	75.5 (10.6)	79.7 (9.2)	**<0.001**
At last MMSE in record	76.9 (9.9)	81.0 (9.0)	**<0.001**
At dementia diagnosis	76.4 (10.5)	80.4 (9.2)	**<0.001**
Other sociodemographic characteristics			
Female (%)	59.9	68.8	**0.047**
Ethnicity (%)			**<0.001**
White	52.5	73.9	
Black	36.9	16.3	
Asian	8.5	7.8	
Other	2.1	2.0	
Married or cohabiting (%)	13.6	30.5	**<0.001**
Mean index of multiple deprivations (SD)	27.8 (9.0)	24.5 (9.6)	**<0.001**
Mean MMSE scores			
1st MMSE	22.0 (6.8)	23.8 (5.5)	**0.008**
2nd MMSE	19.9 (7.3)	20.9 (6.6)	0.186
Last MMSE before dementia diagnosis	17.7 (7.0)	19.8 (6.3)	**0.002**
Last MMSE in record	16.9 (7.2)	18.3 (6.9)	**0.042**
Annual MMSE score decline (95% CI)			
1st to 2nd MMSE	−1.12 (−1.81 to −0.43)	−1.49 (−1.87 to −1.10)	0.143
1st to last MMSE before dementia diagnosis	−1.39 (−1.89 to −0.88)	−1.60 (−1.95 to −1.26)	0.125
1st to last MMSE	−1.24 (−1.61 to −0.87)	−1.70 (−2.00 to −1.41)	0.111
HoNOS Mental health problems^b^ (%)			
Agitated behaviour	14.4	11.0	0.444
Non-accidental self- injury	1.4	7.9	**0.005**
Substance and/or alcohol use	7.3	5.6	0.479
Cognitive problems	36.9	36.5	0.940
Hallucination and/or delusions	53.9	12.7	**<0.001**
Depressed mood	16.3	59.6	**<0.001**
HoNOS Physical health and functional problems^b^ (%)			
Physical illness or disability	45.4	59.8	**0.002**
Problems with activities of daily living	41.8	40.3	0.742
Pharmacotherapy^b^ (%)			
Antidepressant	26.1	75.8	**<0.001**
Antipsychotic	85.9	20.9	**<0.001**
Dementia subtypes (%)			
Alzheimer’s disease	32.4	34.9	0.579
Vascular dementia	25.4	22.2	0.428
Mixed-type dementia (AD and vascular)	19.0	22.0	0.512
DLB	2.8	4.1	0.480
Unspecified or other	20.4	17.3	0.386
Diagnostic services and treatments (%)			
Diagnosis in a memory service	23.9	27.9	0.347
Prior diagnosis of Mild Cognitive Impairment	9.9	18.3	**0.017**
Acetylcholinesterase inhibitor prescribed^c^	32.4	45.4	**0.006**
Memantine prescribed^c^	12.7	21.2	**0.024**

a Mann–Whitney *U* test or chi^2^ test; statistically significant differences highlighted in bold text.

b Around the time of the 1st MMSE recording.

c Anytime in the patient’s record.

### Predictors of dementia in patients with psychotic or affective disorders

Unadjusted Cox regression models and Cox regression models adjusted for age, age squared, gender, ethnicity, marital status, and deprivation score are presented in Supplementary Table 1. In patients with schizophrenia spectrum disorders, only older age at first MMSE and cognitive problems were individually associated with a higher risk of dementia after confounder adjustment, while non-accidental self-injury was associated with a lower risk. In patients with an affective disorder, after confounder adjustment, cognitive and physical health problems and being from a Black or Asian ethnic minority background were associated with a higher dementia risk, whereas higher deprivation and receipt of antipsychotic medication were associated with a lower dementia risk.

### Comparison to the age-matched control cohort

Compared to an age-matched schizophrenia spectrum disorder control cohort who had fewer than two MMSE recordings (*n* = 3,651), the schizophrenia spectrum disorder cohort in whom there were cognitive concerns (*n* = 1,217) showed a four-fold higher dementia risk (20.6% vs. 5.5%), were more likely to be older, Black, have cognitive problems, and be prescribed an antidepressant, but less likely to present with agitated behavior, non-accidental self-injury, and be prescribed an antipsychotic (Supplementary Table 2).

Compared to an age-matched affective disorder control cohort who had fewer than two MMSE recordings (*n* = 6,792), the affective disorder cohort in whom there were cognitive concerns (*n* = 2,264) showed a three-fold higher dementia risk (26.2% vs. 9.5%), were more likely to be older, female and Black, be from a more deprived area, have hallucinations and/or delusions and be prescribed an antipsychotic. They were also less likely to be married and to present with agitated behavior, non-accidental self-injury, depressed mood, physical health, and functional problems (Supplementary Table 3).

## Discussion

Using routinely collected electronic health record data from a large population-based sample, we found that the earliest cognitive concerns in relation to a suspected dementia syndrome, represented by the first recorded MMSE score, arose at a younger age in schizophrenia spectrum, compared to affective disorders (65.0 vs. 71.1 years). The age difference was more pronounced between the schizophrenia and depression groups, where the first MMSEs were recorded 8.9 years earlier in the former. Individuals with schizophrenia or depression who were diagnosed with dementia showed the largest baseline difference in MMSE scores, with the former group scoring 1.8 points lower, although subsequent cognitive trajectories did not differ. Consistent with our prediction, dementia diagnoses were made 2.3 years earlier in schizophrenia spectrum versus affective disorder and 4.0 years earlier in schizophrenia versus depression groups. Our findings align with a recent population-based study reporting an earlier age of dementia diagnosis in schizophrenia compared to bipolar and depressive disorders (Liou, Tsai, Bai, Chen, & Chen, 2022), although the difference between schizophrenia and depression was smaller (around 1.3 years) and diagnosis occurred earlier for both groups (at 64.7 and 66.0 years vs. 76.4 and 80.4 years in our study for schizophrenia and depression, respectively). Other than older age, the presence of cognitive difficulties or non-accidental self-injury at baseline, no other factors significantly predicted dementia risk in individuals with schizophrenia spectrum disorders. We did not detect any differences in the diagnosis of dementia subtypes between cohorts, contrasting with an earlier study (Liou et al., 2022) that found schizophrenia was more strongly associated with unspecified dementia, affective disorders were more strongly associated with AD, and both were similarly associated with vascular dementia.

Both schizophrenia spectrum and affective disorder cohorts are likely to have an earlier age of dementia diagnosis and higher dementia risk than those without psychotic or affective conditions (Liou et al., 2022; Richmond-Rakerd et al., 2022; Stroup et al., 2021). Contrary to our expectations and earlier reports (Liou et al., 2022; Richmond-Rakerd et al., 2022), we found a lower dementia risk in the schizophrenia spectrum versus affective disorders (adjusted HR: 0.81; 95% CI: 0.69–0.95; *p* = 0.010) and in the schizophrenia versus depression (adjusted HR: 0.71; 95% CI: 0.58–0.88; *p* = 0.002) groups. This was despite lower cognitive performance at baseline in patients with psychotic disorders and is unlikely to be explained by different rates of cognitive decline, as annual MMSE change was comparable between cohorts who were diagnosed with dementia. It is possible that our older sample comprised a relatively higher proportion of patients who developed late-onset depression, which has been associated with a higher (particularly Alzheimer’s disease (Robinson et al., 2021) dementia risk, compared to early-onset depression (Singh-Manoux et al., 2017). As individuals with schizophrenia spectrum disorders (Chang et al., 2011), who were 65 years old at baseline, have a shorter life expectancy than other mental health conditions, fewer individuals surviving over the study period to receive a dementia diagnosis may also have contributed to the relatively lower dementia incidence in this group. In addition, healthcare system-related factors or diagnostic and assessment challenges could also have contributed to a lower dementia diagnosis rate in schizophrenia spectrum disorders. For example, after initial cognitive concerns in relation to a possible dementia syndrome were identified, patients with schizophrenia and related disorders received a diagnosis around 6 years later, whereas patients with depression and other affective disorders did so sooner, around 4 years later. Patients with schizophrenia and schizophrenia spectrum disorders were also more likely to live alone and lack a reliable informant. Assessment and treatment challenges may disproportionately impact patients with schizophrenia, as a lower proportion of these individuals received a previous diagnosis of MCI or were prescribed anti-dementia medications compared to those with depression.

Our findings correspond to results from an earlier study (Friedman et al., 2001), which reported an age-related increase in cognitive decline of up to 1 MMSE point per year in institutionalized schizophrenia patients aged between 50 and 80 years. Our study suggests that the oldest individuals (aged 75–80) with the largest cognitive decline rate of 1 MMSE point per year in the earlier study would likely be diagnosed with dementia. It has been suggested that this group shows an age-related pattern of cognitive decline that is different from, and lies between, rates of decline in normal aging or AD (Friedman et al., 2001). One possible explanation is that the schizophrenia spectrum cohort included a proportion (20% in our study) with dementia, of whom a proportion (around a third in our study) were diagnosed with AD. In addition, our observation that cognitive trajectories in schizophrenia spectrum and affective disorders were statistically indistinguishable does not support the concept of a distinct pattern of cognitive decline in schizophrenia and schizophrenia spectrum disorders. As cognitive decline in schizophrenia has not been strongly linked to higher levels of AD pathology (Purohit et al., 1998; Wilson et al., 2024), in contrast to depression symptom severity (Robinson et al., 2021, this supports the concept that these individuals are closer to crossing a clinical threshold that warrants a dementia diagnosis (Kirkpatrick et al., 2008). The relative contributions of proposed ‘primary’ (related to schizophrenia-specific neurobiological/neurophysiological alterations) and ‘secondary’ (related to other source issues) sources of cognitive impairment to later life cognitive trajectories and dementia risk in individuals with schizophrenia are also unclear and may provide an interesting perspective for future studies (Vita et al., 2024).

### Other limitations

As our sample was limited to those with at least two MMSE scores, our findings may have been influenced by selection (inclusion or exclusion) bias. Some individuals who experienced cognitive impairment and/or decline may not have been suspected of having dementia and were not screened with the MMSE. Alternatively, some individuals in whom there were cognitive concerns may not have completed at least two MMSE scores. Our finding that our study sample had a 3–4-fold higher dementia risk compared to age-matched controls supports the concept that the MMSE was generally employed in response to cognitive concerns in relation to suspected dementia risk. There were other differences between patients with at least two MMSE scores and age-matched controls without, for example, study subjects in the former group were more likely to be slightly older and Black. The latter may represent multiple intersecting factors, including historical, social, economic, and healthcare-related issues, so it is unclear whether our study populations were fully representative of the wider patient populations. Future similar studies that compare cognitive trajectories between schizophrenia spectrum or affective disorders with the general older adult population would also be informative.

It is unclear what could underlie a higher annual rate of cognitive decline in the depression versus schizophrenia groups in whom there were cognitive concerns, when no corresponding difference was seen between the groups in individuals later diagnosed with dementia. These sub-group sensitivity analyses involved smaller sample sizes, with the depression sample being approximately three times larger than the schizophrenia sample, which may have limited the reliability and power of these analyses. As our study period was up to 12 years, we cannot exclude the possibility that reverse causation contributed to a proportion of shorter-term associations between later-life mental health disorders and subsequent dementia (Richmond-Rakerd et al., 2022; Tapiainen, Hartikainen, Taipale, Tiihonen, & Tolppanen, 2017). We assumed that the first (and second) MMSE scores represented the earliest cognitive concerns relating to a possible dementia syndrome in individuals, which is supported by the observation that patients were older (over 65 years) at baseline and MMSE recordings occurred earlier in psychotic compared to affective disorders. However, it is possible this assumption was not true in a proportion of individuals, which may have influenced the results. Cognitive assessment via MMSE scores forms only one aspect of dementia assessment and monitoring, and it is possible that alternative or additional measures could provide a more informative picture of cognitive and functional trajectories. While our models were adjusted for prescribed antipsychotic and antidepressant medications, we did not include data on medication dosage or the overall anticholinergic burden of each participant (Joshi et al., 2021), which may have influenced cognition. Finally, our use of electronic health records means that our findings were dependent on the accuracy and quality of data entries. Although we excluded schizoaffective disorder to reduce the potential for diagnostic overlap, we cannot exclude the possibility that some psychotic or affective conditions were misdiagnosed.

Overall, older patients with schizophrenia spectrum and affective disorders who were diagnosed with dementia show similar cognitive trajectories, but initial cognitive concerns relating to a possible dementia syndrome arise from around 63 years in schizophrenia—9 years earlier compared to depression. There is a need for targeted dementia prevention and treatment strategies for these individuals with higher dementia risk and to address existing inequity in dementia assessment and treatment. Further research is also required to explore the potential impact of evidence-based treatment approaches (Vita et al., 2022) on the long-term cognitive trajectory in individuals with schizophrenia spectrum disorders.

## Supporting information

Liu et al. supplementary material Liu et al. supplementary material

## Supplementary material

The supplementary material for this article can be found at http://doi.org/10.1017/S0033291725101864.
