# Supplementary material for: Cognitive trajectories and dementia risk in patients with schizophrenia spectrum versus affective disorders
Source: Psychol Med. 2025 Sep 30;55:e286. doi: 10.1017/S0033291725101864 (PMC12527496; doi:10.1017/S0033291725101864)
Supplement: Liu et al. supplementary material [file S0033291725101864sup001.docx]

**Supplementary online content**

Cognitive trajectories and dementia risk in patients with schizophrenia spectrum versus affective disorders. Kathy Y. Liu, Gayan Perera, Robert Howard, Christoph Mueller.

[Supplementary Table 1: Predictors of dementia in those with schizophrenia spectrum or affective disorders 2](#_Toc169608896)

[Supplementary Table 2: Characteristics of patients with schizophrenia spectrum disorder and ≥2 MMSE scores compared to an age-matched control cohort 3](#_Toc169608897)

[Supplementary Table 3: Characteristics of patients with affective disorder and ≥2 MMSE scores compared to an age-matched control cohort 4](#_Toc169608898)

### **Supplementary Table 1: Predictors of dementia in those with schizophrenia spectrum or affective disorders**

Values shown are hazard ratios (95% CI) for unadjusted models or models adjusted for age, age squared, gender, ethnicity, marital status, deprivation score. Predictors were ascertained around the time of the 1^st^ MMSE recording. Statistically significant (p<0.05) are highlighted in bold.

|  | **Schizophrenia spectrum (n=1,217)** | | **Affective disorder (n=2,264)** | |
| --- | --- | --- | --- | --- |
|  | Unadjusted model | Adjusted model | Unadjusted model | Adjusted model |
| **Sociodemographic characteristics** | |  |  |  |
| Age at 1^st^ MMSE score (per one-year increase) | **1.08 (1.06-1.09)** | **1.19 (1.07-1.33)** | **1.07 (1.06-1.08)** | 1.09 (1.00-1.18) |
| Female gender | **1.87 (1.44-2.43)** | 1.17 (0.89-1.53) | **1.19 (1.00-1.42)** | 0.96 (0.80-1.16) |
| Ethnicity |  |  |  |  |
| White | 1 (reference) | 1 (reference) | 1 (reference) | 1 (reference) |
| Black | 0.85 (0.65-1.10) | 1.00 (0.76-1.31) | 1.01 (0.81-1.27) | **1.41 (1.11-1.78)** |
| Asian | 0.87 (0.53-1.43) | 0.99 (0.60-1.64) | 1.25 (0.92-1.70) | **1.56 (1.14-2.14)** |
| Other | 0.58 (0.21-1.57) | 0.90 (0.33-2.45) | 0.84 (0.44-1.58) | 1.36 (0.72-2.55) |
| Married or cohabiting | **1.46 (1.06-2.02)** | 1.31 (0.94-1.82) | 1.07 (0.90-1.29) | 1.05 (0.86-1.27) |
| Index of multiple deprivations (per one-point increase) | 0.99 (0.98-1.00) | 1.00 (0.98-1.01) | **0.98 (0.97-0.99)** | **0.99 (0.98-1.00)** |
| **HoNOS Mental health problems** | |  |  |  |
| Agitated behavior | 0.71 (0.50-1.01) | 0.76 (0.53-1.08) | **0.76 (0.60-0.97)** | 0.86 (0.67-1.11) |
| Non-accidental self-injury | **0.31 (0.10-0.96)** | **0.32 (0.10-1.00)** | **0.68 (0.49-0.94)** | 0.80 (0.58-1.12) |
| Substance and/or alcohol use | **0.43 (0.25-0.75)** | 1.20 (0.67-2.14) | 0.73 (0.52-1.04) | 1.31 (0.91-1.90) |
| Cognitive problems | **1.31 (1.01-1.71)** | **1.60 (1.22-2.10)** | **1.52 (1.28-1.81)** | **1.69 (1.41-2.03)** |
| Hallucination and/or delusions | 1.17 (0.91-1.51) | 0.95 (0.73-1.23) | 0.97 (0.77-1.22) | 1.19 (0.93-1.51) |
| Depressed mood | 0.98 (0.70-1.36) | 1.12 (0.80-1.57) | 0.92 (0.78-1.09) | 1.01 (0.85-1.20) |
| **HoNOS Physical health and functional problems** | | |  |  |
| Physical illness or disability | **1.35 (1.05-1.73**) | 1.11 (0.86-1.43) | **1.46 (1.24-1.73)** | **1.40 (1.18-1.67)** |
| Problems with activities of daily living | 0.93 (0.72-1.20) | 1.12 (0.86-1.46) | 1.15 (0.97-1.36) | 1.18 (0.99-1.40) |
| **Pharmacotherapy** |  |  |  |  |
| Antidepressant | 0.93 (0.71-1.22) | 1.10 (0.82-1.46) | 1.01 (0.85-1.21) | 1.02 (0.85-1.23) |
| Antipsychotic | 0.87 (0.64-1.17) | 1.10 (0.81-1.49) | **0.57 (0.47-0.68)** | **0.72 (0.60-0.87)** |

### **Supplementary Table 2: Characteristics of patients with schizophrenia spectrum disorder and ≥2 MMSE scores compared to an age-matched control cohort**

|  | **Schizophrenia spectrum & ≥2 MMSE (n=1,217)** | **Schizophrenia spectrum & <2 MMSE (n=3,651^1^)** | **p-value^2^** |
| --- | --- | --- | --- |
| Developed dementia (%) | 20.6 | 5.5 | **<0.001** |
| Age at index date^3^ (mean, SD) | 65.0 (14.8) | 64.1 (14.5) | **0.002** |
| **Sociodemographic characteristics** | |  |  |
| Female (%) | 54.2 | 53.8 | 0.810 |
| Ethnicity |  |  | **0.007** |
| White (%) | 50.1 | 54.2 |  |
| Black (%) | 40.0 | 34.7 |  |
| Asian (%) | 7.2 | 8.6 |  |
| Other (%) | 2.7 | 2.5 |  |
| Married or cohabiting (%) | 14.7 | 15.6 | 0.182 |
| Index of multiple deprivations (mean, SD) | 27.2 (9.1) | 26.8 (9.7) | 0.084 |
| **HoNOS Mental health problems^4^** | |  |  |
| Agitated behaviour (%) | 19.7 | 23.2 | **0.014** |
| Non-accidental self- injury (%) | 3.1 | 4.5 | **0.043** |
| Substance and/or alcohol use (%) | 9.8 | 8.3 | 0.121 |
| Cognitive problems (%) | 27.6 | 20.4 | **<0.001** |
| Hallucination and/or delusions (%) | 57.9 | 58.3 | 0.789 |
| Depressed mood (%) | 17.7 | 16.5 | 0.332 |
| **HoNOS Physical health and functional problems^4^** | |  |  |
| Physical illness or disability (%) | 43.6 | 41.1 | 0.127 |
| Problems with activities of daily living (%) | 39.5 | 38.1 | 0.397 |
| **Pharmacotherapy^4^** |  |  |  |
| Antidepressant | 30.6 | 26.1 | **0.003** |
| Antipsychotic | 79.1 | 84.6 | **<0.001** |

^1^ 1-in-3 matched according to 5-year age-bands

^2^ Mann-Whitney U test or chi^2^ test; statistically significant differences highlighted in bold text

^3^ Index date for the ‘2 MMSE’ cohort was date of first MMSE; index date for the control cohort was recording of first schizophrenia spectrum diagnosis

^4^ closest to index date; for pharmacotherapy +/- 6 months of index date

### **Supplementary Table 3: Characteristics of patients with affective disorder and ≥2 MMSE scores compared to an age-matched control cohort**

|  | **Affective disorder & ≥2 MMSE (n=2,264)** | **Affective disorder & <2 MMSE (n=6,792^1^)** | **p-value^2^** |
| --- | --- | --- | --- |
| Developed dementia | 26.2 | 9.5 | **<0.001** |
| Age at index date^3^ (mean, SD) | 71.1 (12.0) | 70.4 (12.3) | **0.001** |
| **Other Sociodemographic characteristics** | |  |  |
| Female (%) | 62.6 | 57.4 | **<0.001** |
| Ethnicity |  |  | **<0.001** |
| White (%) | 75.7 | 78.5 |  |
| Black (%) | 15.3 | 11.2 |  |
| Asian (%) | 6.7 | 6.3 |  |
| Other (%) | 2.3 | 3.9 |  |
| Married or cohabiting (%) | 29.2 | 32.6 | **0.003** |
| Index of multiple deprivations (mean, SD) | 25.8 (9.8) | 24.3 (10.3) | **<0.001** |
| **HoNOS Mental health problems^4^** | |  |  |
| Agitated behaviour (%) | 15.5 | 18.4 | **0.003** |
| Non-accidental self- injury (%) | 9.5 | 11.9 | **0.002** |
| Substance and/or alcohol use (%) | 7.7 | 9.0 | 0.068 |
| Cognitive problems (%) | 31.3 | 21.7 | **<0.001** |
| Hallucination and/or delusions (%) | 14.5 | 12.8 | **0.047** |
| Depressed mood (%) | 55.5 | 65.1 | **<0.001** |
| **HoNOS Physical health and functional problems^4^** | |  |  |
| Physical illness or disability (%) | 54.9 | 60.8 | **<0.001** |
| Problems with activities of daily living (%) | 39.8 | 42.7 | **0.019** |
| **Pharmacotherapy^4^** |  |  |  |
| Antidepressant | 70.2 | 71.2 | 0.364 |
| Antipsychotic | 33.7 | 30.8 | **0.010** |

^1^ 1-in-3 matched according to 5-year age-bands; note slightly significant difference in mean age despite matching

^2^ Mann-Whitney U test or chi^2^ test; statistically significant differences highlighted in bold text

^3^ Index date for the ‘**≥**2 MMSE’ cohort was date of first MMSE; index date for the control cohort was recording of first schizophrenia spectrum diagnosis

^4^ closest to index date; for pharmacotherapy +/- 6 months of index date
